# CHARM, a gender equity and family planning intervention for men and couples in rural India: protocol for the cluster randomized controlled trial evaluation

**DOI:** 10.1186/s12978-016-0122-3

**Published:** 2016-02-20

**Authors:** Jennifer Yore, Anindita Dasgupta, Mohan Ghule, Madhusadana Battala, Saritha Nair, Jay Silverman, Niranjan Saggurti, Donta Balaiah, Anita Raj

**Affiliations:** Center on Gender Equity and Health, Division of Global Public Health, University of California, San Diego School of Medicine, San Diego, CA USA; National Institute for Research in Reproductive Health, Mumbai, India; Population Council, New Delhi, India; National Institute of Medical Statistics, New Delhi, India; Bill and Melinda Gates Foundation (formerly of Population Council), New Delhi, India

**Keywords:** Male engagement, Family planning, Contraception, Global health, Reproductive health, India

## Abstract

**Background:**

Globally, 41 % of all pregnancies are unintended, increasing risk for unsafe abortion, miscarriage and maternal and child morbidities and mortality. One in four pregnancies in India (3.3 million pregnancies, annually) are unintended; 2/3 of these occur in the context of no modern contraceptive use. In addition, no contraceptive use until desired number and sex composition of children is achieved remains a norm in India. Research shows that globally and in India, the youngest and most newly married wives are least likely to use contraception and most likely to report husband’s exclusive family planning decision-making control, suggesting that male engagement and family planning support is important for this group. Thus, the Counseling Husbands to Achieve Reproductive Health and Marital Equity (CHARM) intervention was developed in recognition of the need for more male engagement family planning models that include gender equity counseling and focus on spacing contraception use in rural India.

**Methods/Design:**

For this study, a multi-session intervention delivered to men but inclusive of their wives was developed and evaluated as a two-armed cluster randomized controlled design study conducted across 50 mapped clusters in rural Maharashtra, India. Eligible rural young husbands and their wives (*N* = 1081) participated in a three session gender-equity focused family planning program delivered to the men (Sessions 1 and 2) and their wives (Session 3) by village health providers in rural India. Survey assessments were conducted at baseline and 9&18 month follow-ups with eligible men and their wives, and pregnancy tests were obtained from wives at baseline and 18-month follow-up. Additional in-depth understanding of how intervention impact occurred was assessed via in-depth interviews at 18 month follow-up with VHPs and a subsample of couples (*n* = 50, 2 couples per intervention cluster). Process evaluation was conducted to collect feedback from husbands, wives, and VHPs on program quality and to ascertain whether program elements were implemented according to curriculum protocols. Fidelity to intervention protocol was assessed via review of clinical records.

**Discussion:**

All study procedures were completed in February 2015. Findings from this work offer important contributions to the growing field of male engagement in family planning, globally.

**Trial registration:**

ClinicalTrial.gov, NCT01593943

## Background

Despite having the oldest national family planning program in the world, India continues to contend with poor rates of contraception use, with almost half of married women of childbearing age reporting no use of modern contraception [1]. Further, modern contraception use that does occur is predominantly in the form of sterilization, which is offered for free and with some additional monetary incentive by the government. As a result, only one-fourth of the total contraceptive users practice modern spacing methods such as pills, condoms, injectables and intrauterine devices [1]. These low rates of modern spacing contraception use drive high rates of unplanned pregnancy [2], inadequate birth spacing, and maternal and infant mortality in the country [1, 3–6]. Spacing contraceptive use is lowest among rural young couples in India [1]. In fact, family planning programs often attempt to reach the young wives only after they reach their ideal family size, despite indications that demand for contraceptive use to delay first pregnancy [7]. Programs to support existing spacing contraception use for young couples in rural India are vitally important for maternal and child health in the region, but remain rare.

Since their inception in 1952, Indian family planning programs and public health initiatives have focused primarily on women (especially through the use of family planning centers, and front-line community health workers such as ASHAs—introduced in 2005), but these programs are meeting with little success in shifting norms to promote spacing contraceptives [8–13]. These family planning programs have paid little attention to gender-based constraints in decision-making. Several research studies show that husbands are key decision makers and often control family planning and family size, especially among rural young couples [1, 7, 14]. Young wives, in particular, are more likely to have husbands who have greater control over and offer less support for contraception use. In addition, low contraceptive knowledge and marital communication and negotiation capacities in this context severely impede likelihood of contraceptive use, even when women would prefer it [7].

Engagement of men is highly recommended to help contend with the gender issues [15] described above, and has shown some success in diverse national contexts, including Nigeria, Malawi, Guatemala, the Philippines, El Salvador, and India [16–22]. However, few interventions exist to engage men in family planning programs in rural India. Of the three documented male-inclusive family planning programs in India that demonstrated improvements in contraception use [20–22], only one involved rural couples. In addition, although that program offered educational outreach to women, men and mothers-in-law, it had difficulty reaching men [22]. None of these interventions involved rigorous randomized controlled trial evaluation. Nonetheless, change over time analyses did document improvements in contraception use, suggesting the potential utility of male engagement family planning approaches for India. Highest rates of male participation came from the clinic-based Men in Maternity study conducted in Delhi [20], suggesting that more structured clinical intervention approaches may be more useful for reaching men than the community education outreach efforts used by the other two studies [21, 22].

Despite the relative success of these male-engagement interventions in improving family planning use, they generally have not been designed to alter the male gender role ideologies that reinforce male reproductive control. Substantial evidence documents that male intimate partner violence intersects with male reproductive control to impede women’s contraception use [23–26]. Hence, targeting male gender inequity ideologies and related partner violence behaviors is increasingly recognized as a key element for male-centered interventions in several health contexts, including promotion of contraception use [15, 18]. Although clinical provider-delivered gender equity counseling for men has not been evaluated, such an approach with women has been proven effective in promoting contraception use in women [27].

With recognition of the need for more male engagement family planning models that include gender equity counseling and focus on spacing contraception use in rural India, the **C**ounseling **H**usbands to **A**chieve **R**eproductive Health and **M**arital Equity (CHARM) intervention, a multi-session intervention delivered to males alone, but included a session with their wives was created. This paper provides an overview of the methods used to develop and evaluate the CHARM study, conducted in rural Maharashtra, India. Findings from this work offer important contributions to the growing field of male engagement in family planning, globally.

## Methods

**Fig. 1:**
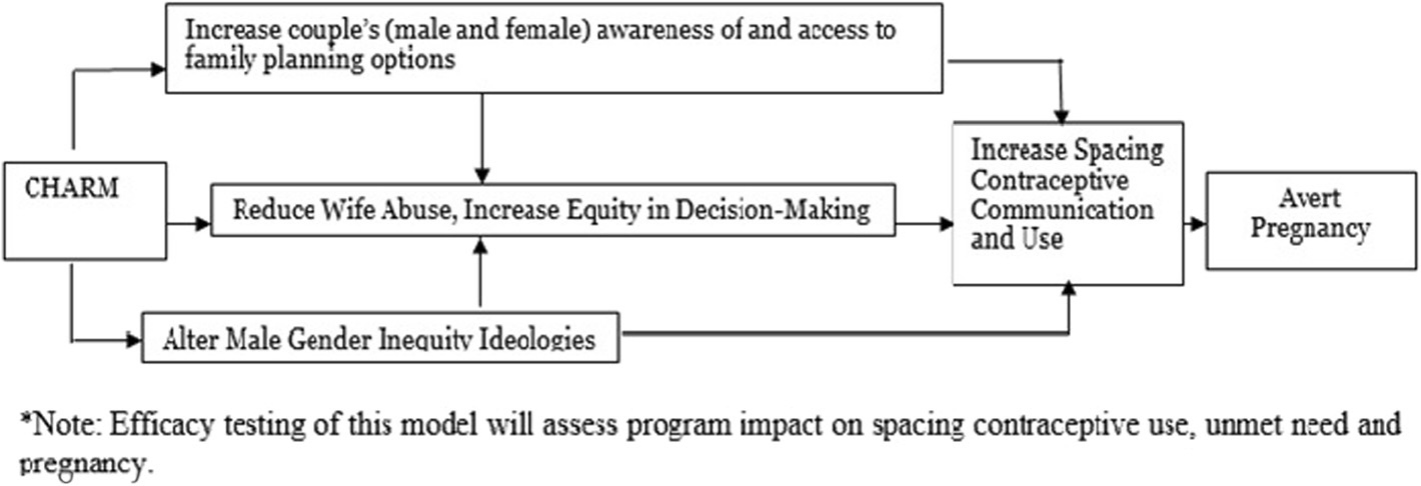
Model of CHARM intervention impact on contraceptive use and pregnancy

This study was designed to develop and test the CHARM intervention, a three session gender-equity (GE) focused family planning (FP) program delivered to married men (Sessions 1 and 2) and their wives (Session 3) by village health providers (VHPs) in rural India. The study involved two phases: 1) development and refinement of the CHARM Intervention and 2) implementation and evaluation of the CHARM Intervention. Phase 1 included formative qualitative research with married men and women, mothers-in-law and health providers in our rural study area for feedback on intervention concepts and approach, as well as community mapping of the area for use in the planned cluster randomization scheme intended for the evaluation trial. The intervention was developed based on existing public health materials and findings from formative research, and pilot tested with a small number of couples and providers to obtain feedback on the intervention and evaluation protocol instruments. Phase 2 involved the implementation and evaluation of CHARM, using a two-armed randomized controlled trial design conducted across 50 mapped clusters randomized to receive either CHARM or the control program (standard FP referral to government public health centers providing FP services), to assess treatment impact on spacing contraceptive use and pregnancy [See Fig. 1]. A process evaluation was also undertaken via interviews with study participants and VHPs and clinical record review, to assess program adherence, participation rates, response to program and ease of delivery.

### Setting

This study was conducted in three Public Health Centers (PHCs) (state government-owned rural healthcare facilities) including Parol, Varjreshwari, and Bathane, located in the Vasai Taluka of Thane District, in Maharashtra, India. A taluka is an administrative division of a region that consists of a city headquarters and a large number of villages. Vasai has 277,262 rural residents comprised largely of Maharashtrian tribal people living in over 250 villages. Vasai, like much of rural India, is characterized by high rates of adolescent marriage and childbirth, low family planning use, and higher maternal and infant morbidity and mortality. The Vasai PHC data, revealed that 95 % of rural young couples who seek family planning services opt for female sterilization, while the remaining 5 % seek spacing family planning services. Analysis of PHC family planning patients by village shows higher spacing contraceptive use among those from villages closer to the PHC, suggesting the importance of family planning access for spacing contraception. The Vasai PHCs are affiliated with Topiwala National Medical College & Bai Yamunabai Laxman Nair Hospital, our collaborating institutions for this project, who facilitated public health partnership for this study.

### Phase 1: intervention development and pilot testing (year 1 of study)

#### Formative research

To inform development of protocols, research tools and training manuals for the CHARM Intervention, formative research was conducted in the form of in-depth interviews with rural young husbands (*n* = 30), rural young wives (*n* = 20), and health providers serving these populations (*n* = 12, 8 private providers and 4 public health providers); and focus groups with mothers-in-law of rural young husbands (*n* = 40 mothers, 4 groups, 8–14 individuals/group). In-depth interviews were used to capture more sensitive and personal information from participants, whereas focus group discussions were used to capture normative attitudes and behaviors within rural families [28]. This formative work was designed to offer comprehensive exploration of the cultural, social, psychological, and family norms and behaviors related to family planning and gender roles and ideologies within marriage from the perspective of rural young husbands, their wives and mothers, and public and private health providers. Findings were used to develop and tailor the proposed intervention to make it culturally-appropriate and locally acceptable, identify optimal methods of capturing key outcome data in terms of language used and survey structure and approach, and support more tailored messaging to intervention participants. Trained Masters-level psychologists and social workers conducted data collection and analysis, under the supervision of senior scientists on the study.

Eligible rural young husbands (*n* = 15 husbands per village) and eligible rural young wives (*n* = 10 wives per village) were randomly selected from the eligible couples’ lists already available via health workers serving each of the two designated study villages. Although not exhaustive, the lists included individuals engaged with the public health system and who were likely more accessible in a shorter timeframe. Eligible rural young husbands and wives were those aged 18–30 years, currently married and regularly residing with their spouse for the past three months, and reporting no sterilization for themselves or their wife. Husbands or wives who were cognitively impaired or in very poor health were to be excluded from participation, but no participants were excluded for this reason. Husbands and wives were not taken from the same household in order to reduce participant’s discomfort when discussing sensitive topics in interviews, in order to address participant’s fears that their spouse might learn of the nature of the discussion between research staff and participant. For both men and women, identified households were visited no more than three times for recruitment attempts. Research staff screened 43 women, of which 37 were eligible and 20 agreed to participate in the study (54 % participation rate). Fifty men were screened, and of these 42 were eligible and 30 agreed to participate (71 % participation rate). Health provider participants (*n* = 12) were invited from the full listing (collected by the research team) of 25 private and public health providers serving the villages of focus (48 % participation rate). For mother in-law focus group participants, key stakeholders from the village (e.g., village leaders, health providers participating in the in-depth interview study) recommended households where mothers of rural young husbands (18–30 years) could be recruited, and stakeholders that recommended mothers-in-law helped research staff recruit them into the study. Of the 67 mothers invited to participate, 40 agreed (60 % participation rate). Rolling recruitment was implemented for all groups until designated recruitment goals were reached. This work was conducted in a single village that was excluded from the larger intervention trial.

Identified participants were screened privately (in the home or nearby) to ensure eligibility, and subsequent in-depth interviews or focus groups were conducted immediately after eligibility was verified and written informed consent was acquired. All data were collected in Marathi, though some providers offered some data in English or Hindi. For men, interviews included questions on; gender attitudes and norms around family planning and reproductive health service utilization, norms of spacing methods of contraceptive use, and contraceptive and fertility practices within marriage, as well as gender equity norms (e.g., IPV acceptability, son preference, child marriage), decision-making and violence within the family, how such norms influence family planning decision-making, and how these norms can be altered with a male-centered intervention on family planning and prevention of partner violence. Women’s interviews assessed; awareness of family planning methods, husbands’ attitudes towards family planning, communication and relationship dynamics with husband, gendered issues and ideologies analogous to those discussed with husbands, as well as how her gender ideologies relate to those of her husband, the impact of her husband’s gender ideologies on partner violence and family planning, and mechanisms through which men could possibly change their behaviors and become positively involved in family planning processes. VHPs and mothers-in-law were also asked similar questions, but at the level of community norms rather than direct experiences. Feedback on optimal recruitment and retention strategies for young married men and their wives was also explored. After the interview, husband and wife participants were given information about PHC family planning programs.

Interview and focus group data were collected using notes rather than audio recordings due to community indications of discomfort with audio recordings. Notes were then detailed fully, translated and typed in English into a Word document. These notes were labeled with the date of the interview/focus group, and the interviewer’s initials. Notes were then reviewed with the supervising scientist, discussed with the interviewer and modified as needed for clarity. Upon completion of full data collection, the research investigator team reviewed all data. Codes were then developed inductively and iteratively, by coders subsequent to actual coding of data, using a grounded theory approach, in which there is a continuous interplay between data collection and analysis to iteratively generate themes and adapt interview guides [29–31]. All codes were designed to be mutually exclusive, but possibly linked. All data were independently coded by two Masters-level trained coders using Atlas.ti v5.0, a computer-based text search program that allows multiple codes to be searched and linked simultaneously [32]. When new codes were identified by coders, they met to agree upon the new codes, introduced them to the research investigator team with example coded data, and if all agreed, added the new codes to the list. They would then return to previously coded data to ensure newly identified codes were coded across all interviews and focus groups. If coders did not agree, a senior level research investigator made final decisions on coded material; this senior investigator also periodically reviewed coding in process to monitor data analysis for quality control purposes. Coded qualitative data were then used to guide intervention development in terms of content and approach, refine concepts for inclusion in the evaluation survey, and guide optimal recruitment and retention strategies for the intervention.

#### Community mapping

Under the direction of scientific leadership in India, the geographic maps generated by the local primary health centers were utilized to identify geographic clusters for the study. Although the original design involved randomization of villages, village size and density varied too much for that to be a reasonable option. Hence, mapping was conducted by Masters-level research staff to identify areas that had comparable population density (approximately 300 households or 1000 population) and somewhat comparable geographic size, with natural boundaries noted to help guide area configurations. Mapping procedures also included indicating public and private health sector facilities as well as community resources and business areas, to ensure clusters were somewhat comparable on these features, as well. This approach resulted in identification of 62 clusters within our study area of focus. Two clusters were randomly selected for pilot testing, and another 50 were randomly selected for inclusion in the larger evaluation study. *Note: Mapping procedures also helped with identification of providers with interest participating in CHARM implementation.*

#### Creation of the CHARM curriculum

CHARM was created using the standard public health family planning counseling guide [33], as well as our theoretical framework [see details on theoretical framework in section on CHARM Intervention Overview] and findings from the formative work. Development was led jointly by the scientific investigator team, a group with experience and expertise in development, adaptation and evaluation of interventions in the areas of gender equity, family planning and sexual health. The ffollowing steps were used to create the intervention and to ensure that it was gender, culturally, and contextually tailored appropriately:

1. An outline of the intervention structure, strategies, and content was created, maintaining core elements of the public health system’s family planning counseling protocol and the gender equity elements guided by the theoretical framework and formative research findings.
2. A scripted curriculum (with graphics) and CHARM VHP training guide was created from the outline developed in Step 1. The curriculum provided details regarding session objectives, goals and activities. Drafted manuals were reviewed and revised iteratively by the full team.
3. Process and outcome evaluation materials were created based on final program objectives and approaches, and previously validated measures and tools, when possible.
4. The scripted manual, training guide and evaluation materials were reviewed and revised based on feedback from formative research and ongoing review by the investigator team, project staff and key informants (rural young married men and women, VHPs, and public health and medical academics familiar with family planning services).
5. All materials were finalized and translated for VHP training and pilot testing.

#### Training of VHPs in CHARM

A total of 22 local VHPs were trained for CHARM delivery, with an initial training session lasting for three days and two half-day refresher trainings over a period of three months focused on the issues of gender-equity focused communication on family planning. All VHPs in the study villages were male, with an average age of 31.6 years (SD = 7.7). The average number of years of practice in the villages was 6 years. Half of providers (11 out of 22) practiced *homeopathic* medicine, two practiced *ayurvedic* medicine (system of traditional Hindu medicine), and the remaining nine providers were allopathic medical providers. Eight VHPs were with the government’s public health system, while 14 were private health practitioners. Of the 22 VHPs trained in CHARM, 21 of them participated in the intervention delivery as part of this study, covering only the 25 intervention clusters, not the control clusters, to reduce risk for contamination. Trainings were provided by public health and academic physicians from Nair Medical University and demographic and behavioral scientists with the government reproductive health research institute (National Institute for Research in Reproductive Health (NIRRH) under the administrative control of Indian Council of Medical Research, Government of India, implementing agency of research activities). Trainings focused on assessment of client’s FP knowledge and goals, the need for male involvement in family planning, safe motherhood and family life, and education on various sexual and reproductive health issues. After the initial CHARM training, the VHPs who did not otherwise have it received FP authorization from TN Medical College (a public health hospital and our collaborator on intervention oversight and implementation), to allow all trained VHPs to provide FP counseling and services, including distribution of condoms and pills. Private VHPs were linked with the public health system to receive free condoms and pills for distribution to CHARM participants. This structure created a public-private partnership to support the intervention. Importantly, FP provision by VHPs was designed NOT to detract from the existing public health model of community outreach for family planning which focuses on outreach to women, but not men. In fact, providers were trained to link with these community health outreach workers (known as ASHAs) to facilitate reinforcement of FP messaging to couples. Three months subsequent to training, a booster training was conducted, focused on gender equity components of the intervention based on feedback that these elements were less understood by the providers.

#### Pilot testing of CHARM and evaluation tools

Pilot testing was conducted to obtain feedback (comprehension, sensitivity, ease of use) on the CHARM model, recruitment and data collection procedures, and evaluation instruments. After development of all CHARM curriculum, evaluation materials and, training of pilot VHPs and research staff, the intervention was pilot tested via implementation and simple pre-post assessment in the two clusters identified for pilot testing in the community mapping procedure described above. Public Health Center maps of the selected clusters were used to identify households with married couples with husbands aged 18–30 years who had not received surgical sterilization via public health services. Households were then randomly selected for recruitment on an ongoing basis until 10 couples per cluster were recruited. Trained NIRRH teams of one male and one female approached the households for screening and recruitment. Married men aged 18–30 years, residing with their wife, and reporting no sterilization of wife or self, were invited to participate in our study. Couples reporting sterilization or who had been diagnosed with infertility (either male or female) were excluded. Participants were screened for eligibility and surveyed via paper surveys as well as via brief open-ended assessments at the end of each survey section, to obtain feedback on survey items and administration. A pregnancy test was also obtained from female participants. Subsequent to data collection, research staff linked male participants to the CHARM-trained VHP for program receipt, with supported follow-up for additional sessions. Transportation support was provided if requested. Three months following baseline, couples and VHPs were contacted to provide feedback via interviews on their perceptions of the CHARM program model. Couples also completed the follow-up survey. Findings were used to finalize all materials and procedures for the efficacy trial. No major changes to curriculum and evaluation procedures occurred, except to provide all core information in Session 1, to allow Sessions 2&3 to be optional, given concerns related to VHP difficulties around following up with men and couples.

### CHARM intervention overview

As noted above, CHARM involved three gender, culture and contextually-tailored family planning and gender equity counseling sessions delivered by trained village health care physicians (VHPs) to men (Sessions 1&2) and couples (Session 3) in a clinical setting, or if required, near or in the home of the participant. (Curriculum available upon request.) The intervention was designed to allow Sessions 2 and 3 to be optional, such that all key information was delivered to the male participant in the first session but extended and reinforced in the subsequent sessions. A desk-sized CHARM flipchart provided pictorial information on family planning options, barriers to family planning use, including gender equity-related issues and the importance of healthy and shared family planning decision-making, to be used by VHPs during male one-on-one and couple-based sessions. VHPs were selected to include private as well as public practitioners, based on private VHPs’ greater availability in the villages. This approach of using VHPs was based on the premise of extending public health services (e.g., family planning supervision/training and contraception options) via the private VHPs, in order to support public-private partnerships for family planning delivery so that learnings would be useful for the national program. As most private VHPs are male, the CHARM intervention was designed to involve men reaching men to improve family planning, and simultaneously, offers more local family planning access. Sessions were to be clinic-based, but near or at home sessions were offered; 59.1 % (n/n = 205/469) of CHARM participants received at least one session at home. All sessions included provision of FP + GE counseling and services. The role of VHPs was to assess and communicate with participants on family planning for a maximum of three sessions (if men expressed interest), provide condoms (to those men who expressed interest), and provide oral contraceptive pills if the couple or the woman presents to the VHP and expressed an interest. The VHPs’ role also included providing a referral to the public health centers for utilization of other contraceptive methods. The private VHPs under the CHARM study were paid an amount of 50 Indian rupees (approximately US$ 0.75) per CHARM session provided to a participant, as compensation for their time and services.

#### Theoretical framework

The CHARM intervention was built on Social Cognitive Theory (SCT) [34] and Theory of Gender and Power (TGP) [35]. SCT is a commonly used theory for effective family planning interventions [36] and posits that improvements in behavior, in this case contraception use, are more likely if a couple perceives positive outcomes for engaging in the behavior (e.g., birth spacing will produce healthier children), feel capable of engaging in and controlling the behavior (e.g., contraceptive knowledge and skills building), and have an environment supportive of the behavior (e.g., access to services) [34]. TGP is a social-structural theory that recognizes the role of power dynamics inherent to many heterosexual dyadic relationships advantaging men over women with regard to sexual and reproductive decision-making and economic and mobility control over issues like contraception acquisition, and use of violence and support from families and communities to maintain that control [35].

### Phase 2: CHARM evaluation (years 2–5 of study)

#### Evaluation study design

**Fig. 2:**
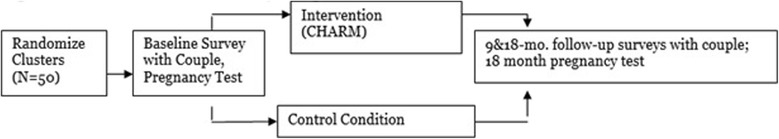
Outcome evaluation study design

Evaluation of CHARM involved a two-armed cluster randomized controlled trial design comparing participants receiving CHARM (VHP-delivered FP + GE over 3 sessions) to those receiving standard of care public health supported family planning services. For outcome evaluation purposes, survey assessments were conducted at baseline and 9&18 month follow-ups with eligible men and their wives, and pregnancy tests were obtained from wives at baseline and 18-month follow-up (See Fig. 2). Impact on spacing contraceptive use and pregnancy were evaluated. [*Unmet need was an original outcome of the study, but standard definitions of this measure changed, and the measures we had included in this study did not allow for standard measurement of this outcome.*] To provide additional in-depth understanding of how the intervention produced any impact observed, VHPs and a subsample of couples (*n* = 50, 2 couples per intervention cluster) participated in in-depth interviews at 18 month follow-up. A process evaluation was also conducted to obtain feedback from husbands, wives and VHPs on their response to the program and fidelity to design, i.e., the extent to which the program elements were implemented according to curriculum protocols, the former based on interviews and latter based on review of clinical records.

#### Random assignment of clusters and cluster preparation for study

Using computer-generated random numbers, clusters selected in the community mapping procedure described previously were randomized by our research team to either intervention or control conditions. Random allocation of clusters into intervention and control conditions group was done on 20th Feb 2012 at 2:05 PM. A schedule was then created to roll out the study for recruitment over a one year period, on a rolling basis. In the month prior to roll out of the study in each selected cluster, intervention and control, the study team met with key providers and leaders in that community to provide a more supportive infrastructure for study implementation. Group meetings were held that involved village panchayat leaders (recognized and respected community elders serving as village leaders), ASHAs (community health outreach workers) and other village-level public health workers (auxiliary nurse midwives—ANMs) to discuss the project and how it will be implemented. ASHAs were also engaged to connect to CHARM VHPS and to provide linkage to CHARM couples for public health family planning services, when requested.

#### Participants and eligibility criteria

Primary study participants for intervention evaluation were rural young men and their wives. Inclusion and exclusion criteria are identified in Table 1, below.

**Table 1:** Criteria for participation of couples

Inclusion criteria	Exclusion criteria
Age 18–30 years and Fluent in Marathi	Cognitive Impairment (husband or wife)
Willing to Have Wife Included in the Study	Sterile or Wife is Sterile
Residing in the village for the past 2 years and residing with wife in village for past 3 months	Intend to move in next 18 months or either spouse refuses participation

Surveys were administered at baseline and 9&18 month follow-ups, pregnancy tests were taken at baseline and 18 month follow-up to determine program impact on key outcomes (spacing contraceptive use, communication). A subsample of CHARM couples (*n* = 50, 2 couples per CHARM intervention cluster) participated in in-depth interviews at 18 month follow-up to assess how the program affected their relationship and behaviors. Male CHARM participants also completed a brief survey on their perceptions of the program. Qualitative data were also collected from CHARM VHPs regarding their views of the program at study completion to provide VHP perceptions on how the intervention impacted family planning behaviors. Between March 2012 and December 2012, 1,881 couples were screened and 1,043 were determined to be eligible to participate in the study. Of those identified as eligible, 1,081 couples were enrolled into CHARM and completed a baseline survey. A log was maintained to track eligibility, participation and enrollment rates by cluster and treatment group, reasons for refusal among the eligible, and dates of approach and screening. These were reviewed on weekly calls to track the recruitment and enrollment process for quality and scheduling purposes, and make course corrections as needed.

#### Procedure

Immediately subsequent to household screening and recruitment, eligible husbands and their wives were asked by trained Masters-level research staff to participate in the baseline survey (measures detailed below). Immediately subsequent to obtaining written informed consent, the survey was administered on paper for each spouse separately in a private location, by a gender-matched NIRRH staff member. Following the women’s survey, women were asked to take a urine pregnancy test under the direction of the research staff member. Test results were available within three minutes of testing and were noted by the interviewer on the woman’s survey. The interviewer confirmed the test results with the women and provided information on local antenatal services as well as family planning services at the PHC, as appropriate. Husbands were not notified of wives’ pregnancy testing or test results. After the men’s baseline survey, males in the intervention condition were notified that family planning counseling is now available in the village via trained VHPs as part of CHARM, and supported referral (i.e., direct linkage and transport if needed) to the VHP was provided. Participants were reminded that additional data would be collected from them in 9&18 months. Research staff followed up with both VHPs and men to ensure linkage occurred and to track sessions delivered. Women from both intervention and control conditions were provided subsequent to baseline survey detailed information regarding public health FP services, and linked to community health outreach workers (ASHAs) if requested. No monetary incentive was provided for study or program participation.

**Fig. 3:**
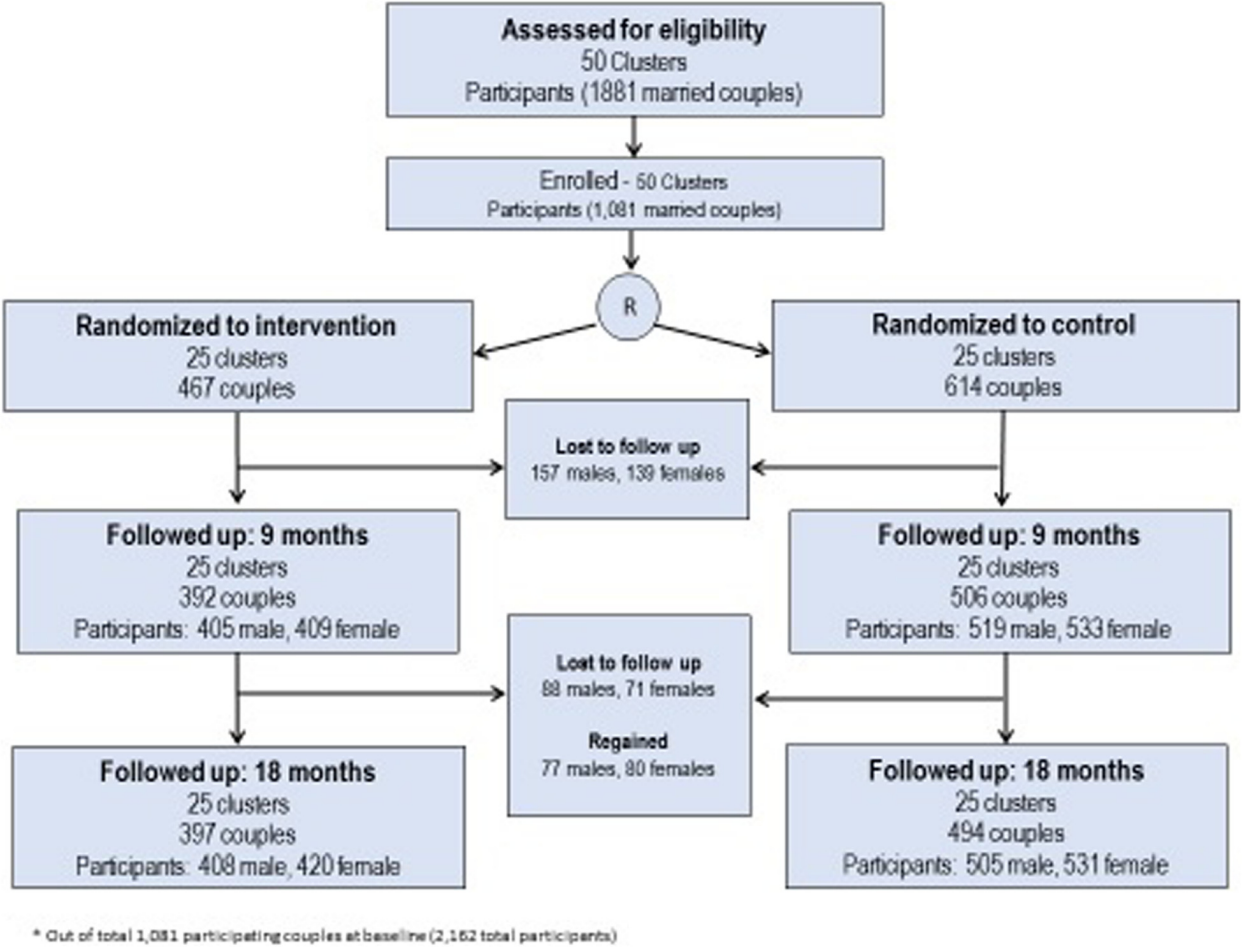
Consort flowchart

The three VHP-based CHARM intervention sessions were to be completed within a three month timeframe for all intervention arm participants. Most men recruited from CHARM communities (91.3 %) received Session 1 of CHARM; 77.2 % received Session 2, and 52.5 % received the couple’s session with their wife. Follow-up survey assessments were conducted at 9&18 months post-baseline with couples from both comparison and treatment clusters using the same procedures described above for baseline data collection; no additional referrals were provided at these time points unless requested. Additionally, at 18 month follow-up, wives were tested for pregnancy; pregnancy tests were obtained at only two time points to reduce costs and burden for interviewers. Also at 18 month follow-up, a subsample of couples (two couples per intervention village) were randomly selected to participate in in-depth interviews subsequent to their surveys; interviews assessed how the intervention affected the way the couple considers, discusses and uses family planning methods in their relationship. CHARM-trained VHPs (*n* = 16) participated in brief in-depth interviews subsequent to completion of the study to assess their perceptions of how the intervention affected family planning practices among rural young men and their wives. From March to December 2012, baseline data were collected. Subsequently, 9 month follow-up data collection was collected from November 2012 to September 2013, and 18 month follow-up surveys were collected 9 months after those dates. Of the 1081 couples participating in baseline assessment, 83.1 % (*n* = 898) and 82.4 % (891 couples) completed 9 and 18 month follow-ups, respectively [See Fig. 3. Consort Flowchart.].

Due to the nature of the intervention and study design, neither the study subjects nor the research and program staff were blinded. However, the study was designed to minimize measurement bias. Additionally, all research interviewers were trained on the importance of uniform interview procedures. Drs. Balaiah, Nair and Saggurti were responsible for the training and supervision of these research interview staff.

Qualitative data was collected from a subsample of intervention couples (*n* = 50 couples) and CHARM-trained VHPs (*n* = 16 VHPs) who conducted any sessions, in order to provide further insight into outcome findings. At 18 month follow-ups, 2 randomly selected participating couples from each intervention village (*n* = 50 couples total) were asked to participate in in-depth interviews. The interviews were jointly conducted by the NIRRH team of 1 male and 1 female who also conducted the survey separately with the couple. Only couples who participated in all 3 sessions (including wives in session 3) were eligible for qualitative interview participation, biasing the perspectives on the intervention but allowing for insight into the utility of the full program. The in-depth interview guideline for couples assessed respondents’ perceptions of how their relationship and family planning practices changed since participating in the program and how the intervention created or affected these changes. Interviews were 45–60 min in length and conducted in Marathi. CHARM-trained VHPs who delivered sessions (*n* = 16) also participated in in-depth interviews subsequent to study completion. The 30 min open-ended interviews with intervention VHPs assessed their perceptions of how CHARM affected the couples with whom they met and whether behavior changes and ongoing contact for family planning services continue subsequent to the intervention. They were also asked about the strengths and weaknesses of the program. Data were collected and analyzed using the same methods as described above for formative research.

#### Quantitative measures

Baseline and follow-up surveys were collected from couples using measures based on India’s Demographic and Health Surveys (DHS/India’s NFHS-3) [1] or other validated measures (indicated in Table 2).

**Table 2:** Quantitative measures

Variable	Description
Demographics	age, caste, religion, education, literacy, income, employment, and living conditions
Marital Factors	age at and length of marriage, frequency of sexual activity marital communication
Fertility and FP History	age at first pregnancy, number and timing/wantedness of pregnancies and childbirths, sex of children, current breast feeding, FP history (timing and type of contraceptives used).
Fertility/FP normative beliefs and ideologies	ideal number of children, in total and based on sex (parity, son preference); Attitudes toward contraception, contraceptive knowledge, desire for and intent to use traditional and/or modern contraceptives (by type), and perceived access to all forms of contraception
Sexual risk behaviors	past year sexual infidelity or sex work involvement, condom use in these contexts
Male Gender Equity Norms (only men asked)	15 item scale measured male gender norms related to sexual and reproductive health, sexual relations, violence, domestic responsibilities, and homophobia. Gender-Equitable Men Scale [38] (CHARM Cronbach alpha .72);
Male Partner Violence^a^ (only women asked)	11 items assessed physical and sexual marital violence, ever and in past 12 months. (CHARM Cronbach alphas .82–.92 across subscales)
Acceptability of spousal violence	two scales of 8 and 10 items assessed reasons for acceptability of spousal physical and sexual violence. CHARM Cronbach alpha .82 &. 92, respectively
FP behaviors	item assessed contraception used (e.g., withdrawal, condom, IUD, sterilization, etc.) in the past 3 months. Item assessed discussion of contraceptive use with spouse.
Pregnancy and Pregnancy Intent	data on current pregnancy was obtained via a urine test for human chorionic gonadotropin (HCG) at baseline and 18 month follow-up. Survey data also captured self-reported pregnancy and whether they want to become pregnant then, later, or not at all.

^a^Items on spousal violence were only be assessed for women for purposes of her safety in accordance with guidelines for domestic violence research from the World Health Organization [37]

Intervention participants also completed a brief survey at follow-up on their experiences with the intervention, including FP content covered in sessions, delivery of contraceptives, perceptions of the strengths of the VHPs, perceptions of the utility of the men-only and couple sessions, and recommendations for the program. This was part of the process evaluation component to assess quality of delivery and response to program. A weakness of this assessment was lack of measures focused on GE elements of the intervention. For the participant satisfaction surveys, most men assigned to receive CHARM also provided survey responses (84.9 %; n/n = 398/469). Most responders (87.2 %; n/n = 347/398) had participated in CHARM and were able to provide feedback on their experiences of intervention delivery

#### Quantitative data collection, management and analysis

Baseline survey data were collected on paper. Paper surveys were reviewed by research staff immediately subsequent to data collection to ensure completeness. Paper surveys were transported nightly to the local office in rural Vasai, and transported weekly to the data management office in Mumbai, for data entry. For every survey collected, research staff noted date of collection, assigned participant identification number, interviewer initials, and location of data entry, into the survey (Vasai office or transported to Mumbai for data entry). All hard copy surveys were maintained in a locked file in the research offices. Masters-level staff conducted double data entry, with consistency checks and verifications performed on an ongoing basis, to ensure high quality data. Although the plan was for collected data to be entered within a month of collection, in practice, data entry staff were responsible for other project tasks and unable to maintain consistent data entry during baseline data collection. Much of the baseline data were entered over the course of the eight months following completion of baseline data collection. Periodic (bimonthly) reviews of entered data in descriptive format were shared with the study team for quality assurance. The plan was to use Perseus for mobile data collection, but lack of Marathi font impeded our ability to use this software.

For 9&18 month follow-up survey data collection, our study team worked with software engineers at Qualcomm Institute, University of California—San Diego, to create the MSHARE (Mobile Survey for Health Assessment, Research and Evaluation) System, for electronic data collection and web-based data management. This system was designed to display any font in any language. The CHARM surveys were coded using XML into the android operating system-based MSHARE System. The MSHARE application with the CHARM survey questionnaire was then loaded onto the tablets, displaying questions simultaneously in English and Marathi, to help ensure clarity of the questions for our bilingual research staff. MSHARE captured data more accurately than paper surveys, and allowed for uploading of data to a web-based data management system for real time data sharing, such that the US and Indian investigator teams had access to data simultaneously from any location with internet access. Each field researcher maintained their own android tablet to collect and store data, short-term. The memory available in tablets allowed the field researcher to save hundreds of completed surveys for a 25–30 page survey. At the end of each day of data collection, the field researcher brought their tablets to a field research site to upload the data via internet. Lack of Wi-Fi availability in rural Vasai impeded immediate upload in the field, though MSHARE allowed for this capacity had Wi-Fi been available. Conversion to MSHARE allowed 9 month-follow-up data collection completed in October to be presented at a conference in November. Uploaded data were monitored weekly for assurance of transfer into the MSHARE database, and data were analyzed monthly and presented to the research team for quality review and discussion.

#### Labeling and tracking data

All data collected in the study, whether on paper or tablet, were labeled with a unique identifier to de-identify the data but allow linkage of longitudinal data from the same subject and data from husbands and wives. Identifiers were linked with participant contact information to support tracking and linkage, but kept in a locked file at the local research office in India, to facilitate follow-up by the field team, as needed. All labeling and tracking information, minus identifiers, were kept in a log with a schedule of study assessments populated, and notification of date of data collection at each time point. This was monitored and tracked daily by the field site supervisor to monitor recruitment and follow-up rates throughout the study. Monthly meetings with VHPs included collection of participant session participation, which was then linked to this monitoring sheet to observe study participation. These data were used for dose analyses in the evaluation. The full monitoring sheet was reviewed on weekly calls between the US and Indian investigator teams throughout the course of data collection for this study.

#### Institutional review board (IRB) approval and ethical treatment of participants

All procedures were reviewed and approved by the IRBs of University of California at San Diego, Population Council and India’s National Institute for Research in Reproductive Health. NIRRH led oversight of research procedures in the field to monitor and ensure ethical treatment of participants and adherence to study protocol. This study was registered at clinicaltrials.gov (NCT01593943).

A data and safety monitoring plan was also in place and focused on two major safety aspects: 1) assurance that no harm came to participants as a result of survey or program participation and 2) assurance that all data collected from this project, including process and quality assurance data as well as outcome data, would maintain the privacy of research participants. To ensure no harm, and with recognition of high rates of domestic violence in India, at the time of each survey data collection, participants were assessed for immediate health and social service needs in the areas of FP, maternal or antenatal health, and violence, by research staff trained to provide referrals and links to health and social services, in accordance with the WHO guidelines for ethical research on domestic violence [37]. Participants were also notified via informed consent that their withdrawal from the program would not affect their participation in the survey participation and vice versa. Also, withdrawal from both the program and the survey participation would have no impact on the services they received at the local health clinic or within their work organization. Through informed consent, participants were additionally notified of research staff they could contact should any difficulties arise through their participation in this project. We did not anticipate that the surveys or medical record reviews would cause harm to enrollees, but the IRBs from all partnering institutions involved in this study (NIRRH, ICMR, Population Council and UCSD) had strict reporting requirements for adverse events and a yearly reporting process with which we complied with at all times. Multiple efforts were made across all study phases to reduce risks for adverse events. However, a data and safety monitoring plan was used to identify such events if they did occur. The plan was as follows: Should an adverse event be identified, the principal investigators from each site were to be notified within 48 h, and then there was to be a discussion that week whether the adverse event had anything to do with the research study (e.g., a death due to subject’s occupation), or whether it occurred as a consequence of an individual’s study participation (e.g., abuse of a participating wife subsequent to intervention involvement). IRBs and funders were to be notified immediately following the discussion. If it were the latter, considerations of how to reduce such risks in the future were to be determined and protocols were to be altered accordingly. No adverse events occurred, so the plan never required operationalizing.

Data security and confidentiality were also a priority of this study. As noted above, all data were de-identified, and participant identifiers such as name or location were maintained on separate forms in locked storage at a different location from the analytical database. Only field staff and their supervisor, a member of the scientific team, had access to the linked information. Data shared between investigators across countries were maintained on secure servers that only allow connections from users authenticated from the domain controller. Password protection also locked individual data fields. In terms of data management, all hard data collected from this study was brought to a field office for data entry, coding, management and analysis. Hard data safety was assured by locking the collected data, consent forms, contact numbers and addresses in separate securely locked cabinets in a locked office that was only accessible to the field supervisor. Electronic data files (e.g., survey data collected via tablets) were maintained on secure servers and labeled only by unique identifiers. If any security arrangement had been violated, either through physical tampering or breach of confidentiality, the individual making the discovery were to notify the Principal Investigators, and it would have been handled as an adverse event, but as noted above, no issues arose. We believe the risks to the subjects were reasonable in relationship to the anticipated benefits.

#### Sample size calculation

Sample size and power considerations were constructed based on our primary outcomes of interest: any spacing contraceptive use and communication. As noted above, unmet need was an original outcome of the study, but our item measures did not allow for an accurate assessment of this variable. A baseline sample size of 1000 couples, equally distributed between the 50 clusters/villages, as well as equally distributed between the intervention and the control groups was assumed. We also assumed 20 % attrition (at the end of 18-months of follow-up) and thus, based our calculations on 800 men. All calculations were based on 2-sided logistic regressions with a significance level of 0.05. While we utilized longitudinal regression methods in our analyses, for the purposes of power calculations we utilized single time-point methods. However, considering general longitudinal analyses yield higher power, this should not be a problem. Furthermore, due to the cluster randomization of the proposed study, our computations were adjusted for the design effect in order to account for the correlation of subjects within the same village. Assuming 20 men enrolled in each village, and a within village correlation (Kappa) of 0.10, the design effect [1 + within village correlation*(# of men per village-1)] was estimated to be equal to 2.9. We should note that the assumed kappa value of 0.10 is within the range of what is often observed in clinical studies, and may be considered a conservative assumption given that we adjusted for potential village level confounders in the regression models. Below, we present power calculations to assess the minimum differences we aimed to detect with 80 % power for the primary endpoints of each outcome. The estimates used for these calculations came from NFHS-3 data on young married rural Maharashtrian men [1].

*Hypothesis 1:* This hypothesis tests the effect of intervention on the use of marital spacing contraceptives. Power calculations were based on Logistic Regression of a dependent binary variable (i.e. use of marital spacing contraceptives) on a binary independent variable (i.e. Group 1 = Intervention Group, Group 2 = Control Group) with 800 subjects equally distributed between the two groups. Referring to the power curves in Fig. 3, if the proportion of men reporting use of contraceptives in Group 2 is 8 %, 10 %, or 12 %, then we will have over 80 % power to detect a difference between Group 1 and Group 2, when the corresponding proportion in Group 1 is 20 % (OR_Group1/Group2_ = 2.8), 22 %(OR_Group1/Group2_ = 2.59), and 25 %(OR_Group1/Group2_ = 2.45), respectively. Based on the NFHS-3 data, we expected that the proportion of men using any marital spacing contraception in the control group (Group 2) would be 10 % at 18 months. Based on the above assumptions, the proposed study had 80 % power to detect an absolute difference as small as 12 % between the two groups. A difference of 12 % is considered clinically meaningful.

**Fig. 4:**
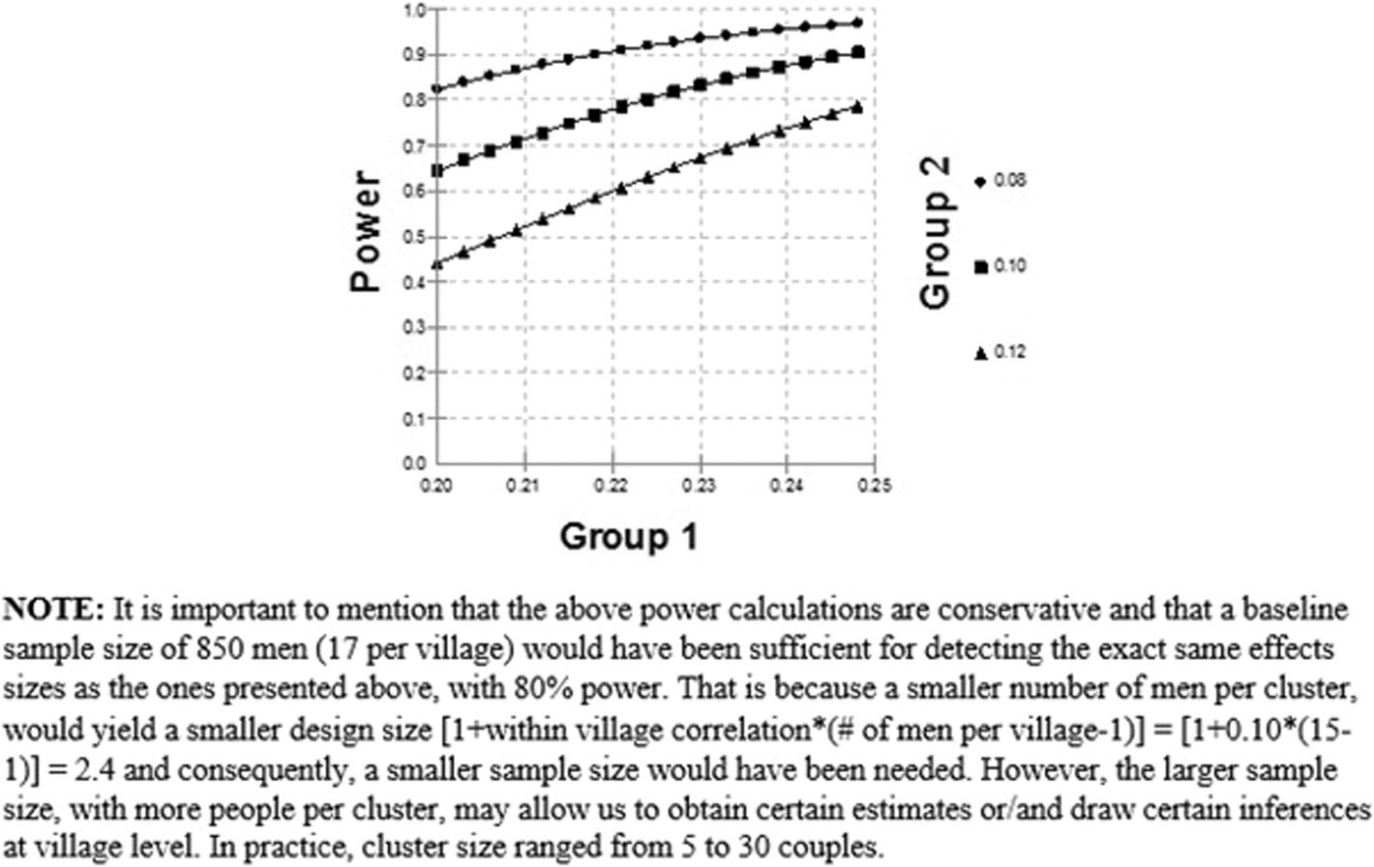
Power to detect the effect of intervention on use of marital spacing

**Fig. 5:**
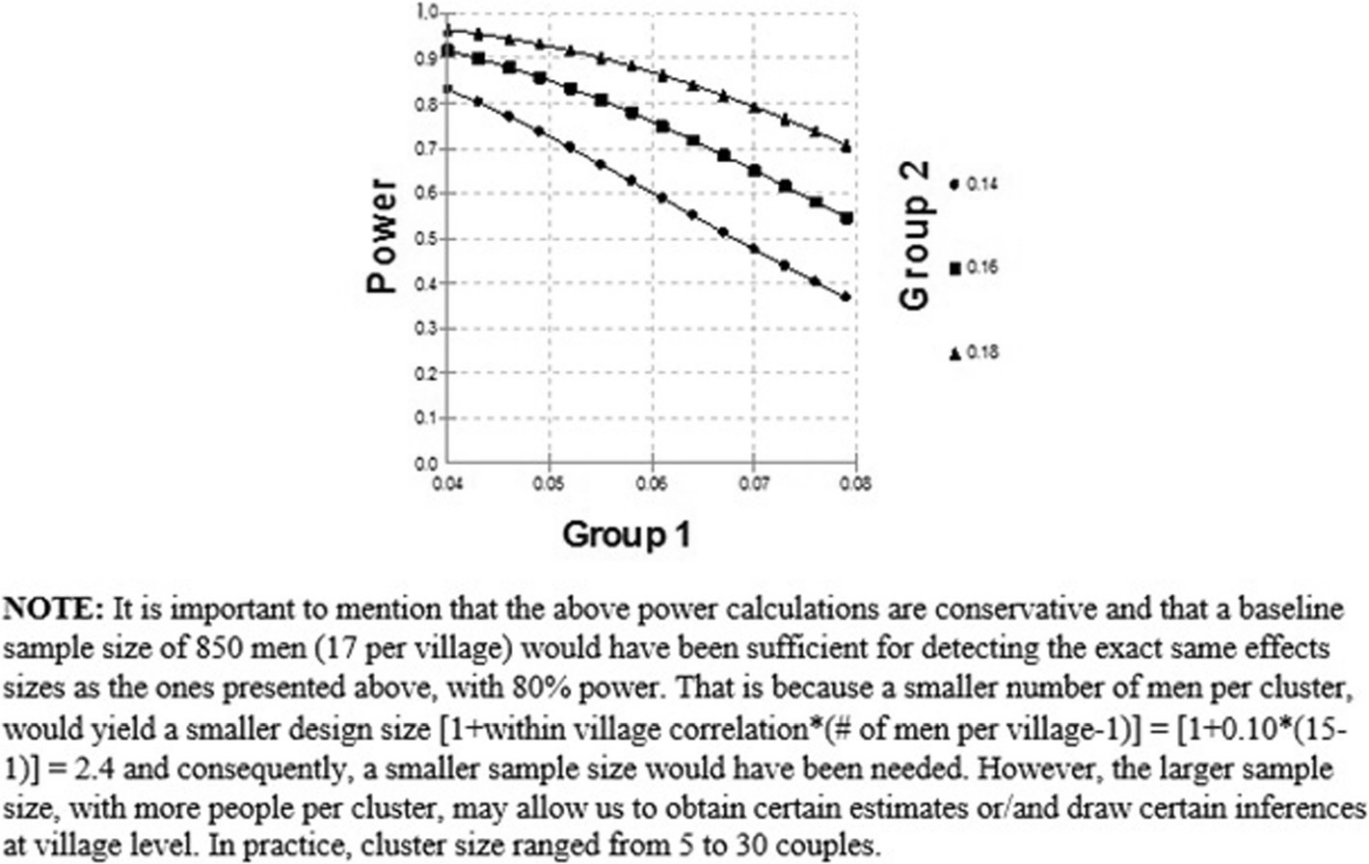
Power to detect the effect of intervention on pregnancy

*Hypothesis 2:* This hypothesis tests the effect of intervention on any pregnancy at 18 month follow-up. Power calculations were based on Logistic Regression of a dependent binary variable (i.e. reporting pregnancy at 18 month follow-up) on a binary independent variable (i.e. Group 1 = Intervention Group, Group 2 = Control Group). Referring to the power curves in Fig. 4, if the proportion of men reporting pregnancy for wives at 18-months in Group 2 is 14 %, 16 %, or 18 %, then we would have over 80 % power to detect a difference between Group 1 and Group 2, when the corresponding proportion in Group 1 is 4 % (OR_Group1/Group2_ = 0.28), 6 % (OR_Group1/Group2_ = 0.31), and 7 % (OR_Group1/Group2_ = 0.34), respectively. Based on the NFHS-3 data, we estimated that the proportion of men reporting any pregnancy for wives at the 18 month follow-up in the comparison group would be 16 %. The proposed study had 80 % power to detect an absolute difference of at least 10 % between the control and intervention groups (Fig. 5).

#### Analytic plan

Outcome analyses were designed to focus on the following outcomes of interest: spacing contraceptive use and communication, and included pregnancy, intimate partner violence and attitude outcomes based on women’s reports and pregnancy testing. The primary comparison of the outcomes by treatment group at 9 and 18 month follow-ups were done using mixed-effects regression models, with a nested random effects structure for the geographical clusters and for each couple within the cluster, over time. The treatment effect was evaluated via a time-by-treatment arm interaction. A categorical time effect was used (profile model). This treatment effect can be interpreted as the individual-level effect of the intervention. In addition, generalized estimating equations (GEE) logistic regression models with the same clustering and mean model structure and exchangeable working correlation matrix was used to express the intervention effect at the population level. Secondary analyses adjusted for relevant covariates to improve precision and possibly reduce bias. Demographic and fertility variables (e.g., age, current pregnancy, past year childbirth, etc.) were considered for inclusion in adjusted analyses, in addition to time and treatment arm, using backward model selection at the 0.15 threshold level. Bivariate analyses were conducted to assess differences in demographics, fertility history, and outcomes at baseline: 1) by treatment group, 2) for those lost to study follow-up, and 3) for CHARM participants lost to intervention. Any characteristics identified as significantly different between groups were considered as potential covariates in these adjusted models. Outcome analyses used an intent-to-treat approach and analyzed all subjects according to randomized group. Additional analyses were conducted based on receipt of intervention. All analyses used women’s data only, but men’s data will be used to validate findings on contraceptive behavior outcomes. All analyses were conducted using SAS (SAS Institute, Version 9.4, Cary, NC, USA).

#### Process evaluation

The process evaluation was designed to ensure quality delivery of the program and to assess response from participants and providers. To measure the quality of delivery and fidelity to program design, we did the following: 1) Provided standard training for all VHPs, as noted above, and conducted pre-post analysis after each session on FP and GE knowledge and attitudes, using these data to address in the next training session any gaps identified, 2) Required checklists and case sheets for each session delivered by the VHP to track session delivery, ensure coverage of all pieces of the intervention (indicated on the checklist), and assess issues that arose in the sessions for follow-up at next session or elsewise (using case notes), 3) Conducted clinical supervisory meetings with all VHPs on a monthly basis to discuss CHARM delivery and specific cases of concern, with review of VHP checklists and case sheets. CHARM VHP supervision was conducted jointly by preventive medicine and FP experts, 4) In-depth interviews with intervention VHPs and select study participants to provide feedback on their view of the intervention, as described above; and 5) Brief surveys conducted with intervention participants at follow-up to assess their perception of FP components of the program and their perceptions of the intervention, as noted above. (See Table 3 for details on each piece of process evaluation and how it was managed and analyzed.) Collecting these data enabled quality control by the research team and pragmatic feedback for the program staff to maintain quality delivery of the intervention across VHPs. Data collection related to this component were used to: a) ensure adherence to the intervention in terms of structure, content, and strategies; b) ensure that staff training and experience relevant to program implementation is consistent with the guidelines set for the intervention; and c) assess staff and subject response to the programs. Protocols and tools for this component were developed for this study.

**Table 3:** Process evaluation components for CHARM project

Form	Who completes form and when	Who collects form and when	Purpose of form	How is data processed
1. VHP Checklist	Who: VHPs	Who: ^b^Data Manager will collect and give to ^b^Scientific Lead in the Field and ^b^Monitoring and Evaluation Manager	Quality control to ensure all activities were covered in each session. A one page checklist for VHPs to use to make sure they cover each piece.	Reviewed by ^b^Scientific Lead in the Field and ^b^Monitoring and Evaluation Manager, monthly; Feedback provided to VHPs in monthly meetings; Update provided to US Team Monthly during weekly conference call; entered into SPSS
- Individual Sessions	When: Just before the conclusion of each intervention session			
		When: Monthly.		
2. VHP Casesheet	Who: VHPs	Who: ^b^Data Manager will collect and give to ^b^Scientific Lead in the Field and ^b^Monitoring and Evaluation Manager	This sheet is for VHPs to assess participant's attitudes, knowledge around the family planning and gender equity issues; and provide necessary communication.	Reviewed by ^b^Scientific Lead in the Field and ^b^Monitoring and Evaluation Manager, monthly; Feedback provided to VHPs in monthly meetings; Update provided to US Team Monthly during weekly conference call; entered into SPSS
- Individual sessions	When: During each intervention session			
		When: Monthly.		
2. Final Participant Satisfaction Survey^a^	Who: CHARM Intervention Participants (male only). Conducted by field team	Field staff, at the end of endline survey (9 months post-baseline)	Participant satisfaction with CHARM intervention	Entered with quantitative data; prevalence data used for reports
	When: At time of endline/9 month survey.			
3. In-Depth Interviews with VHPs	Who: All CHARM VHPs subsequent to program completion. IDIs will be conducted by field team	Field staff, after the 9 months follow-up but before the 18 month follow-up.	Feedback on perceptions of strengths and weaknesses of CHARM in terms of recruitment, retention, intervention delivery and mechanisms of outcome effects.	Transcribed/translated and analyzed by field team using Atlas-ti.
		Note: All VHPs should be interviewed if they provided at least one session.		
	When: Subsequent to completion of study.			
4. In-Depth Interviews with Male Participants and Female Participants	Who: CHARM Intervention Participants only (both male and female participants). 10 % of all participants randomly selected for inclusion **as couples, among those completing all three sessions**.	Field staff, after intervention delivery for the couple is over, at a survey follow-up assessment.	Feedback on perceptions of strengths and weaknesses of CHARM in terms of recruitment, retention, intervention delivery and mechanisms of outcome affects.	Transcribed/translated and analyzed by field team using Atlas-ti.
	When: At 9 or 18 month follow-up.			

^a^ *form is data entered*

^b^Indicates name was removed and replaced with title of position

Additional data: Notes taken at monthly meetings b/w Indian intervention team will be conveyed during Indo-US weekly meetings to check ongoing issues and implementation. Meeting notes from Indo-US meetings will be maintained for review

## Discussion

The CHARM Intervention is a three session gender-equity (GE) focused family planning (FP) program delivered to married men (Sessions 1 and 2) and their wives (Session 3) by village health providers (VHPs) in rural India. It was developed and tested through a scientifically rigorous process to ensure its capacity to provide evidence for the effectiveness of male engagement models of family planning intervention that include gender equity counseling to men. As described in this protocol, formative qualitative research with married men and women, mothers-in-law and health providers in our rural study area was used to provide feedback on intervention concepts and approach, with consideration of our theoretical framework inclusive of both Social Cognitive Theory [34] and the Theory of Gender and Power [35] to create the intervention approach and build upon existing FP counseling materials from the Indian public health system [33]. The area of study was mapped to guide understanding of service availability in the study area and to create a cluster randomization scheme to be used in the evaluation trial. The intervention was developed by the scientific team with feedback from community members and FP and GE experts; the final intervention was manualized in a flip chart form and pilot tested with 20 couples. Final refinements were made to the intervention and then the CHARM program was evaluated using a two-armed randomized controlled trial design conducted across 50 mapped clusters randomized to receive either CHARM or the control program (standard FP referral to government public health centers [PHCs] providing FP services), to assess treatment impact on spacing contraceptive use and pregnancy. A process evaluation was also undertaken via interviews with study participants and VHPs and via clinical record review to assess program adherence, participation rates, response to program, and ease of delivery. The procedures were implemented with no adverse events and with good participation in the CHARM intervention (91.3 % of men received at least one session) and strong evaluation follow-up (>80 % retention at each follow-up).

### Conclusion

This paper reviews the study protocol for the CHARM Intervention, a two-armed cluster randomized controlled design study. The CHARM model is a male engagement family planning intervention, inclusive of gender equity counseling and with a focus on spacing contraceptive use for young couples, in rural India. Findings from this study have the potential to greatly contribute to the field of male engagement in family planning. All study procedures were completed in February 2015.

## Abbreviations

ANM: auxiliary nurse midwife
ASHA: accredited social health activist
CHARM: Counseling Husbands to Achieve Reproductive Health and Marital Equity intervention
DHS: Demographic and Health Survey
FP: family planning
GE: gender equity
GEE: generalized estimating equations
ICMR: Indian Council of Medical Research
IRB: institutional review board
MSHARE: Mobile Survey for Health Assessment, Research and Evaluation
NFHS: National Family Health Survey
NIRRH: National Institute for Research in Reproductive Health
OR: odds ratio
PHC: public health center
SCT: social cognitive theory
SD: standard deviation
TGP: theory of gender and power
VHP: village health provider
